# Patients’ adherence to smartphone apps in the management of bipolar disorder: a systematic review

**DOI:** 10.1186/s40345-021-00224-6

**Published:** 2021-06-03

**Authors:** Marie-Camille Patoz, Diego Hidalgo-Mazzei, Bruno Pereira, Olivier Blanc, Ingrid de Chazeron, Andrea Murru, Norma Verdolini, Isabella Pacchiarotti, Eduard Vieta, Pierre-Michel Llorca, Ludovic Samalin

**Affiliations:** 1 Department of Psychiatry, CHU Clermont-Ferrand, University of Clermont Auvergne, CNRS, Clermont Auvergne INP, Institut Pascal, Clermont-Ferrand, France; 2 Bipolar and Depression Disorders Unit, Institute of Neuroscience, Hospital Clinic, CIBERSAM, University of Barcelona, Barcelona, Catalonia Spain; 3 Fondation FondaMental, Créteil, France; 4 Service de Psychiatrie B, Centre Hospitalier Universitaire, 58 rue Montalembert, 63000 Clermont-Ferrand, France

**Keywords:** Smartphone, Mobile application, Bipolar disorder, Systematic review

## Abstract

**Background:**

Despite an increasing number of available mental health apps in the bipolar disorder field, these tools remain scarcely implemented in everyday practice and are quickly discontinued by patients after downloading. The aim of this study is to explore adherence characteristics of bipolar disorder patients to dedicated smartphone interventions in research studies.

**Methods:**

A systematic review following PRISMA guidelines was conducted. Three databases (EMBASE, PsychInfo and MEDLINE) were searched using the following keywords: "bipolar disorder" or "mood disorder" or “bipolar” combined with “digital” or “mobile” or “phone” or “smartphone” or “mHealth” or “ehealth” or "mobile health" or “app” or “mobile-health”.

**Results:**

Thirteen articles remained in the review after exclusion criteria were applied. Of the 118 eligible studies, 39 did not provide adherence characteristics. Among the selected papers, study length, sample size and definition of measures of adherence were strongly heterogeneous. Activity rates ranged from 58 to 91.6%.

**Conclusion:**

The adherence of bipolar patients to apps is understudied. Standardised measures of adherence should be defined and systematically evaluated in future studies dedicated to these tools.

**Supplementary Information:**

The online version contains supplementary material available at 10.1186/s40345-021-00224-6.

## Introduction

With a lifetime prevalence rate of more than 2% (Merikangas et al. 2007), bipolar disorder (BD) is a common and disabling chronic disease (Pini et al. 2005). BD is characterised by successive mood episodes separated by inter-episodic periods often associated with residual symptoms and poor functioning (Samalin et al. 2014; Murru et al. 2018). The management of BD could be facilitated by the exponential spread of smartphone apps, (Carson et al. 2016) which are easy and useful tools to monitor both subjective and objective mental health status in ecological momentary conditions (Myin-Germeys et al. 2016; Depp et al. 2015; Wenze et al. 2014; Moore et al. 2016) and to provide adjunctive psychosocial interventions (Hollis et al. 2015).

However, despite an increasing number of available mental health apps (Firth et al. 2016) and significant interest from patients (Ben-Zeev et al. 2013) and therapists (Kerst et al. 2019), these tools remain scarcely implemented in everyday practice (Kerst et al. 2019). Furthermore, like any app, mental health apps are quickly discontinued by patients after downloading (Bauer et al. 2020; Torous et al. 2020). A recent review of digital self-help apps or programmes for depression and anxiety reported that 7–42% of participants continued to actively use the app after 4 weeks but only 0.5–28.6% after 6 weeks (Torous et al. 2020). Despite promising results regarding their interest in the treatment of depression (Kerst et al. 2019) and BD (Depp et al. 2015), the failure of smartphone apps to maintain patient adherence over time could be a major barrier to their implementation in mental healthcare. The low number of scientifically validated apps available on app stores, resulting in a lack of trust from users, and the lack of user-centric designs have often been cited as explanations for this low adherence to apps (Torous et al. 2018; Nicholas et al. 2017). Their integration in therapy has also been described as an important factor in adherence, with higher adherence to adjunctive apps being observed than to unguided ones (Eysenbach 2005; Baumeister et al. 2014). Furthermore, most of the studies reporting positive results do not report adherence, leaving an inaccurate measure of patients receiving or not the intervention, for how long and with which frequency. Thus, a placebo effect cannot be discarded.

An under-studied topic in itself, adherence to smartphone apps have nevertheless been evaluated in several reviews in the field of mental health (Ng et al. 2019; Batra et al. 2017) and depression (Torous et al. 2020; Fleming et al. 2018). To the best of our knowledge, this work has not specifically examined BD, nor has any systematic review been published focused on the adherence characteristics of patients to smartphone apps.

### Aims of the study

In this systematic review, we explore the adherence characteristics of bipolar disorder patients to dedicated smartphone interventions in research studies.

### Methods

This review was conducted in accordance with the Preferred Reporting Items for Systematic Reviews and Meta-Analyses (PRISMA). It has been registered in PROSPERO (CRD42020218984).

### Literature search

A search of the electronic databases MEDLINE, PsycINFO and Embase was conducted to identify peer-reviewed English and French language articles published between January 1st, 2008 and August 1st, 2020. We chose not to include articles published prior to 2008 as this was the year the first app store was released. The existence of articles concerning mental health apps before this date seemed unlikely.

The search terms were designed to capture any smartphone intervention targeting BD patients: ("bipolar disorder" OR "mood disorder" or bipolar) AND (digital OR mobile OR phone OR smartphone OR mHealth OR ehealth OR "mobile health" OR app OR mobile-health).

### Study selection process

Articles were included if:

- They included patients with a BD diagnosis
- They involved the delivery of an intervention or collection of data via a smartphone app or a personal digital assistant (PDA), with or without an associated wearable device
- They were randomised or non-randomised trials, with or without a control group, pilot or feasibility studies
- They measured and described the adherence characteristics to the studied app
- They either included or did not include face-to-face therapy in combination with the app intervention

Articles were excluded if:

- They were reviews, commentaries, qualitative studies, conference reports or protocols
- They did not focus on technologies or the disorder of interest (*i.e.* a mobile phone app or PDA and BD)
- They only involved screening or diagnostic tools
- The intervention was only based on SMS
- They discussed data reported in a previous study
- They reported preliminary results only

### Data extraction

We utilised a data extraction template developed for this systematic review for the extraction of the following characteristics and data:

- Article reference details and year of publication
- Study characteristics: study design, primary outcomes, study length and main findings.
- Sample characteristics: sample size, mean age, % of female participants, % of participants with BD type 1, clinical state, mean depression and manic scores at baseline.
- Intervention details: name, version and type of app, type of phone used by participants (personal or loaned), existence of notifications, psychoeducation, collection of passive data and feedback, number of sessions (corresponding to one or more items evaluated by the app through questions or tests) a day and number of items actively evaluated in a session.
- Adherence characteristics: activity rate (number of sessions initiated by the patient on the total number of available sessions during the study; when the study protocol included one session a day, the activity rate was then defined as the number of days the patient started a session on the total number of days in the study); completion rate (number of days with a fully completed session on the total number of days in the study); median or mean of use (median or mean time before the patient discontinues the use of the app); retention rate (number of patients still actively using the app at a given time).

Adherence is a generic term that can encompass several aspects. If studies displayed terms as “mean adherence”, “compliance rate” or “adherence rate” without providing any definition, we wrote to the corresponding authors for more details. Where the definition of these terms remained unclear, we gathered them under the broad term “undefined adherence characteristics”.

Furthermore, to obtain comparable data, we had to discern whether the adherence characteristics were calculated relative to all patients or to active patients only. When this information could not be found in the published paper, we wrote to the corresponding authors for more details. In the event the authors did not reply, we considered that adherence characteristics were calculated relative to all patients, including inactive ones.

When studies included populations with control groups, we selected only data and outcomes regarding BD patients in experimental groups using the studied app.

### Statistical analysis

Statistical analysis was performed with Comprehensive Meta-analysis software (Borenstein et al. 2020). To address the non-independence of data due to studies effect, random-effects model using the method of DerSimonian & Laird (with study as random-effect) was performed to estimate mean and standard-deviation (SD) of age of participants in the included studies taking into account the between- and within-study variability (DerSimonian and Laird 2015).

### Methodological quality assessment

The quality of each included study was assessed by two independent authors (LS and MCP) using the NIH study Quality Assessment Tools (National Institute of Health 2021). The tool used was adapted to each study design (Quality Assessment of Controlled Intervention Studies, Quality Assessment Tool for Observational Cohort and Cross-Sectional Studies and Quality Assessment Tool for Before-After (Pre-Post) Studies With No Control Group) Discrepancies between the two raters were solved by discussion between them until a consensus was reached.

## Results

**Fig. 1:**
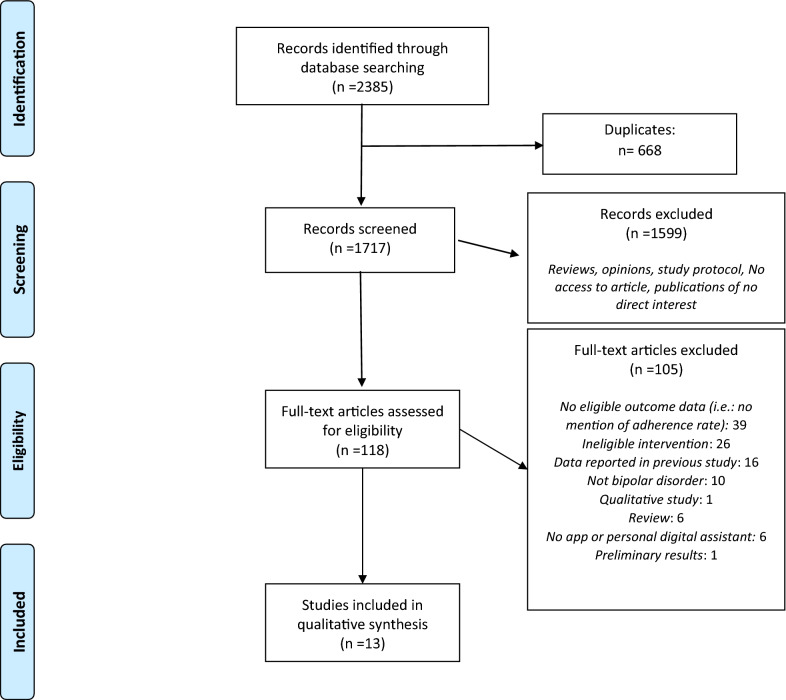
Prisma flowchart

The search retrieved 1717 records after duplicates were removed. Among them, 228 abstracts were assessed for eligibility and 110 were excluded. A full-text review of the 118 remaining articles was conducted. A total of 13 studies were included in the review (Depp et al. 2015; Wenze et al. 2014, 2016; Faurholt-Jepsen et al. 2014, 2015, 2020; Til et al. 2020; Hidalgo-Mazzei et al. 2016, 2018; Beiwinkel et al. 2016; Tsanas et al. 2016; Stanislaus et al. 2020; Schwartz et al. 2016). The most common reasons for exclusion in the full text article stage were that no adherence characteristics to the studied app were found in the article (n = 39) or that a non-eligible intervention was reported (n = 26). Figure 1 is a flow-chart of the considered and ultimately selected studies, following the PRISMA statements.

### Characteristics of included studies and patient populations

The characteristics of the 13 included studies are summarised in Table 1. They were conducted in the United States (Depp et al. 2015; Wenze et al. 2014, 2016; Til et al. 2020; Schwartz et al. 2016), Denmark (Faurholt-Jepsen et al. 2014, 2015, 2020; Stanislaus et al. 2020), England (Tsanas et al. 2016), Spain (Hidalgo-Mazzei et al. 2016), Germany (Beiwinkel et al. 2016) and in spanish language countries (Hidalgo-Mazzei et al. 2018). Among them, three were randomised controlled trials (Depp et al. 2015; Faurholt-Jepsen et al. 2020; Til et al. 2020) and 10 were non randomized studies (Wenze et al. 2014, 2016; Faurholt-Jepsen et al. 2015, 2014; Hidalgo-Mazzei et al. 2016, 2018; Tsanas et al. 2016; Stanislaus et al. 2020; Schwartz et al. 2016). Among the non randomized studies, four were feasibility studies (Wenze et al. 2014, 2016; Hidalgo-Mazzei et al. 2016, 2018) and two were pilot studies (Faurholt-Jepsen et al. 2014; Beiwinkel et al. 2016). Study lengths ranged from 14 to 800 days, with a mean of 213.1 days (SD: 215.3),just over half of the studies (7/13) lasted 6 months or more (Depp et al. 2015; Faurholt-Jepsen et al. 2015, 2020, 2015; Hidalgo-Mazzei et al. 2018; Beiwinkel et al. 2016; Stanislaus et al. 2020).

**Table 1:** Study characteristics

Author, year, location	Study design	Primary outcome	Study length (Weeks)	Main findings	Additional intervention
Van Til et al*.*; 2020, US (Til et al. 2020)	Randomised Control Trial	To identify how to best engage individuals with BD in monitoring their symptoms using mobile and wearable technology	6	No statistical difference in adherence between the two groups (passive and active monitoring)	
Faurholt-Jepsen et al.; 2020, Denmark (Faurholt-Jepsen et al. 2020)	Randomised Control Trial	To assess the effect of smartphone-based monitoring and mood prediction on depressive and manic symptoms in patients with BD	36	No differences between the intervention group and the control group in levels of depressive and manic symptoms	
Depp et al.; 2015; US (Depp et al. 2015)	Randomised Control Trial	To assess the effect of the PRISM programme on depressive symptoms using an app or paper and pencil in patients with BD	24	Significant effect on depressive symptoms, greater in the app group compared to the paper and pencil group	In-person psycho-educational programme associated with the use of the app
Stanislaus et al.; 2020, Denmark (Stanislaus et al. 2020)	Observational	To compare frequency of mood instability between BD patients and healthy control using a smartphone app	Up to 800 days	Mood instability score was statistically significantly higher for patients with BD compared with HC	
Hidalgo-Mazzei et al.; 2018, Spanish language countries (Hidalgo-Mazzei et al. 2018)	Observational, Feasibility study	To evaluate the long-term retention, usability, perceived helpfulness, and satisfaction among the first 201 users of the SIMPLE programme	24	More than 30% of the participants continued to use the programme after 6 months. Positive outcomes regarding satisfaction, usability, and perceived helpfulness	
Tsanas et al.; 2016, UK (Tsanas et al. 2016)	Observational	To introduce and validate a novel clinical questionnaire used for daily mood monitoring of BD and borderline personality disorder patients as part of a smartphone application	12 to 48	Daily MZ items of negative mood correlated highly with the clinical scores. Correlations were weaker between the daily ratings of positive mood clinical scores	
Schwartz et al., 2016, US (Schwartz et al. 2016)	Observational	To measure completion rates of surveys of mood symptoms on smartphone in BD and healthy control participants	2	Median completion rates did not differ between groups	
Wenze et al.; 2016; US (Wenze et al. 2016)	Observational, Feasibility study	To assess feasibility and acceptability of a 12-week adjunctive, smartphone assisted intervention to improve treatment adherence in bipolar disorder	12	Average Credibility and Expectancy Scale total score was 42.13 (SD = 9.64) out of a possible total of 54. Good adherence to the intervention overall. High satisfaction in qualitative feedbacks	In-person psycho-educational programme associated with the use of the app
Hidalgo-Mazzei et al.; 2016; Spain (Hidalgo-Mazzei et al. 2016)	Observational, Feasibility study	To evaluate acceptability, safety, and satisfaction of the simple app	12	The SIMPLe app represents a satisfactory and acceptable instrument as an add-on to the usual treatment	
Beiwinkel et al.; 2016; Germany (Beiwinkel et al. 2016)	Observational, Pilot study	To investigate whether smartphone data predict impending clinical symptoms in bipolar disorder	48	Self-reported mood was found to predict depressive symptom levels above the clinical threshold but not manic symptoms	
Faurholt-Jepsen et al.; 2015; Denmark (Faurholt-Jepsen et al. 2015)	Observational	To determine indicator of illness activity to investigate differences between Bipolar 1 and Bipolar II patients using smartphone self-monitoring	24 or more,	Patients with Bipolar II experienced more severe depressive symptoms and spent almost half of their time with depressive symptoms whereas patients with Bipolar I were euthymic during 75% of their time	
Wenze et al. 2014, US (Wenze et al. 2014)	Observational, Feasibility study	To establish the feasibility and acceptability of using mobile technology to target adherence in BD	2	Participants voiced satisfaction with study procedures. Adherence with EMI sessions was high	
Faurholt -Jepsen et al.*;* 2014, Denmark (Faurholt-Jepsen et al. 2014)	Observational, Pilot study	To investigate possible correlations between clinically rated depressive and manic symptoms of bipolar disorder and subjective and objective smartphone data	12	Increasing depressive symptoms correlated with decreasing amounts of movement per day. No correlation between smartphone measures and manic symptoms identified	

*BD* Bipolar disorder, *HC* Healthy control, *SD* Standard deviation, *EMI* Ecological momentary intervention

The quality assessment of the studies included in this systematic review outlines a wide heterogeneity in studies design, populations and outcomes see Additional File 1, 2, 3, Five studies were rated with poor quality because of the small number of participants enrolled (Wenze et al. 2014, 2016; Faurholt-Jepsen et al. 2014; Beiwinkel et al. 2016; Schwartz et al. 2016). Six studies were rated with fair quality (Faurholt-Jepsen et al. 2015; Til et al. 2020; Hidalgo-Mazzei et al. 2016, 2018; Tsanas et al. 2016; Stanislaus et al. 2020) and 2 were rated with good quality (Depp et al. 2015; Faurholt-Jepsen et al. 2020).

**Fig. 2:**
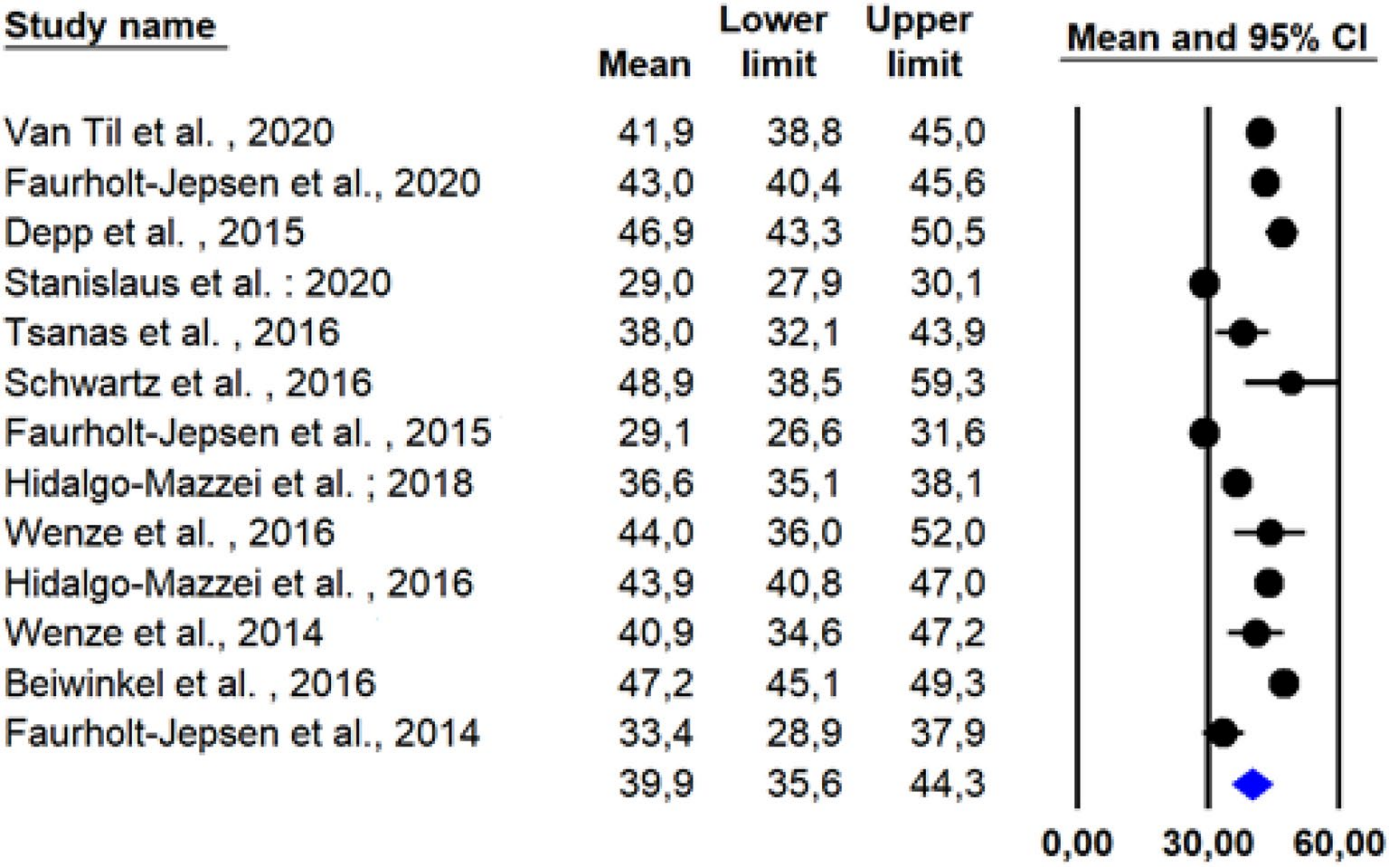
Mean and standard-deviation of age of participants in the included studies taking into account the between- and within-study variability

The characteristics of the included patients are summarised in Table 2. They suffered from type 1, 2 or unspecified BD. One study included participants with at least moderate depression and mania (score  ≥ 11 on the Quick Inventory of Depressive Symptoms (QIDS-C33) and/or  ≥ 16 on the Clinician-Administered Rating Scale for Mania (CARS-M34)) (Wenze et al. 2016), three studies included only euthymic patients, (Depp et al. 2015; Faurholt-Jepsen et al. 2015; Hidalgo-Mazzei et al. 2016) 1 included patients that could be in any mood state at the time of enrolment (Schwartz et al. 2016) and 8 studies did not specify if the participants were euthymic or not (Wenze et al. 2014; Faurholt-Jepsen et al. 2020, 2014; Til et al. 2020; Hidalgo-Mazzei et al. 2018; Beiwinkel et al. 2016; Tsanas et al. 2016; Stanislaus et al. 2020). Participants were predominantly female (n = 481, 62.4%), with a mean age of 39.9 (Inter Quartile Range (IQR): 35.6-44.3,Fig. 2.

**Table 2:** Population characteristics

Reference study	Sample size N	Mean age, Years (SD)	FemaleN (%)	BD type 1 N (%)	Clinical state	Depression score at baseline (SD)	Manic score at baseline (SD)
Til et al. (2020)	47	41.9 (10.8)	25 (53.1)	32 (68.0)	NS	NS	NS
Faurholt-Jepsen et al. (2020)	85	43.0 (12.4)	52 (61.2)	54 (63.5)	NS	NS	NS
Depp et al. (2015)	41	46.9 (11.8)	22 (53.7)	36 (87.8)	Euthymic	MADRS: 11.7 (Moore et al. 2016)	YMRS: 7.4 (Myin-Germeys et al. 2016)
Stanislaus et al. (2020)	203	28.0 [24-35] *	140 (69.0)	68 (33.5)	NS	HAMD17: 9 [5-15] †	YMRS: 2 [0-7]^a^
Hidalgo-Mazzei et al. (2018)	201	36.6 (Firth et al. 2016)	127 (63.2)	NS	NS	NS	NS
Tsanas et al. (2016)	48	38.0 (Ng et al. 2019)	32 (66.7)	NS	NS	NS	NS
Schwartz et al. (2016)	10	48.9 (16.8)	7 (70.0)	10 (100)	Any mood state	NS	NS
Wenze et al. (2016)	8	44.0 (11.6)	5 (62.5)	5 (62.5)	At least moderate depression or mania	QIDS-C 15.2 (3.2)	CARS-M 7.2 (4.3)
Hidalgo-Mazzei et al. (2016)	51	43.9 (11.4)	21 (42.9)	33 (67.3)	Euthymic	HAMD-17: 3.2	YMRS: 2.1 (2.6)
Beiwinkel et al. (2016)	13	47.2 (3.8)	5 (38.5)	6 (46.1)	NS	NS	NS
Faurholt-Jepsen et al. (2015)	33	29.1 (7.4)	23 (71.0)	20 (60.6)	Euthymic	HAMD: 9 [4-16]^a^	YMRS: 2 [0–7]
Wenze et al. (2014)	14	40.9 (12.1)	10 (71.4)	5 (35.7)	NS	QIDS: 12.5 (4.0)	CARS-M: 9.9 (8.7)
Faurholt-Jepsen et al. (2014)	17	33.4 (9.5)	12 (70.6)	14 (82.4)	NS	NS	NS

*BD* Bipolar disorder, *SD* Standard deviation, *NS* Not specified, *MADRS* Montgomery and asberg depression scale, *YMRS* Young mania rating scale, *HAMD17* Hamilton depression rating scale with 17 items version, *HAMD* Hamilton depression rating scale, *QIDS-C* Quick inventory of depressive symptomatology-Clinician Rating, *QIDS-C* Quick inventory of depressive symptomatology, *CARS-M* Clinician administered mania rating scale

^a^Median [interquartile range]

Three studies mentioned retribution for participants, among which 1 paid patients for the time they spent on the study (Depp et al. 2015) and two gave compensation for each session completed (Wenze et al. 2014, 2016).

### Characteristics of interventions

Intervention characteristics are summarised in Table 3. Twelve studies assessed a smartphone app (Depp et al. 2015; Faurholt-Jepsen et al. 2014, 2015, 2020; Wenze et al. 2016; Til et al. 2020; Hidalgo-Mazzei et al. 2016, 2018; Beiwinkel et al. 2016; Tsanas et al. 2016; Stanislaus et al. 2020; Schwartz et al. 2016) and one a PDA (Wenze et al. 2014). All the interventions included self-monitoring sessions. In addition, four apps collected additional passive data (Faurholt-Jepsen et al. 2014, 2020; Til et al. 2020; Beiwinkel et al. 2016), four included the delivery of psychoeducational messages (Wenze et al. 2014, 2016; Hidalgo-Mazzei et al. 2016, 2018) and one included a wearable (Til et al. 2020). Four studies investigated the original or upgraded MONARCA app (Faurholt-Jepsen et al. 2014, 2015, 2020; Stanislaus et al. 2020), two investigated the original or upgraded SIMPLe app (Hidalgo-Mazzei et al. 2016, 2018) and the remaining seven studies investigated an original intervention (Depp et al. 2015; Wenze et al. 2014, 2016; Til et al. 2020; Beiwinkel et al. 2016; Tsanas et al. 2016; Schwartz et al. 2016). Regarding self-monitoring, all the studies offered sessions once- (Faurholt-Jepsen et al. 2014, 2015, 2020; Hidalgo-Mazzei et al. 2016, 2018; Beiwinkel et al. 2016; Tsanas et al. 2016; Stanislaus et al. 2020) or twice-a-day (Depp et al. 2015; Wenze et al. 2014, 2016; Til et al. 2020; Schwartz et al. 2016). The Moodzoom and SIMPLe apps (original or upgraded) added a weekly complementary session (Hidalgo-Mazzei et al. 2016, 2018; Tsanas et al. 2016). The mean number of evaluated items was 7.5 (SD: 4.0). Two interventions evaluated only two items (Beiwinkel et al. 2016; Stanislaus et al. 2020) and five evaluated ten or more items (Wenze et al. 2014, 2016; Faurholt-Jepsen et al. 2014, 2015, 2020). The most frequently evaluated items were mood (12 studies (Depp et al. 2015; Wenze et al. 2014, 2016; Faurholt-Jepsen et al. 2014, 2015, 2020; Til et al. 2020; Hidalgo-Mazzei et al. 2016, 2018; Tsanas et al. 2016; Stanislaus et al. 2020; Schwartz et al. 2016), irritability, anxiety or stress (nine studies (Wenze et al. 2014, 2016; Faurholt-Jepsen et al. 2015, 2020, 2014; Hidalgo-Mazzei et al. 2016, 2018; Tsanas et al. 2016; Schwartz et al. 2016)), sleep [seven studies (Wenze et al. 2014, 2016; Faurholt-Jepsen et al. 2015, 2020, 2014; Hidalgo-Mazzei et al. 2016, 2018)] and medication adherence [seven studies (Wenze et al. 2014, 2016; Faurholt-Jepsen et al. 2014, 2015, 2020; Hidalgo-Mazzei et al. 2016, 2018)]. Nine interventions provided notifications or SMS reminders to complete self-monitoring sessions (Depp et al. 2015; Wenze et al. 2014, 2016; Faurholt-Jepsen et al. 2015; Hidalgo-Mazzei et al. 2016, 2018; Beiwinkel et al. 2016; Stanislaus et al. 2020; Schwartz et al. 2016). Eight interventions provided feedback on self-monitoring through graphics or messages (Depp et al. 2015; Wenze et al. 2014, 2016; Faurholt-Jepsen et al. 2015, 2020; Til et al. 2020; Hidalgo-Mazzei et al. 2016, 2018).

**Table 3:** App characteristics

App (study)	Version	Type	Phone	Notifications	Psychoeducation (Type)	Collection of passive data (Type)	Feedback (Type)	Sessions in a day	Number of items evaluated in a session	Adherence Characteristics
No Name (Til et al. 2020)		Self-monitoring + wearable (Fitbit)	Personal	No	No	Yes (Physical activity, sleep and heart rate measured with a wearable device)	Yes (Graphical feedback)	2^a^	6	Completion Rate: 81.8%
MONARCA (Faurholt-Jepsen et al. 2014)	Original	Self-monitoring	Loaned	No	No	Yes (speech duration, social activity, physical activity, and cell tower ID)	No	1	10	88.0%^b^
Faurholt-Jepsen et al. 2015)	Upgraded	Self-monitoring	Loaned	Yes	No	No	Yes (Clinical feedback loop)	1	11	93.0%^b,c^
Faurholt-Jepsen et al. 2020)	Upgraded	Self-monitoring	Personal or loaned	No	No	Yes (Phone usage, Social activity, Physical activity, Mobility)	Yes (Clinical feedback loop)	1	10	Activity rate: 72.6%
Stanislaus et al. 2020)	Upgraded	Self-monitoring	Personal or loaned	Yes	No	No	No	1	2	Median of use: 107 days (IQR 157)
PRISM (Depp et al. 2015)		Self-monitoring and evidence-based intervention	Loaned	Yes	No	No	Yes (Graphical Feedback)	2^a^	9	Activity rate: 65.0%
Moodzoom (Tsanas et al. 2016),		Self-monitoring	NS	No	No	No	No	1	6	Activity rate: 81.9%^c^
No Name (Schwartz et al. 2016)		Self-monitoring	Loaned	Yes	No	No	No	2	5	Activity rate: 79.0%
Myexperience (Wenze et al. 2016)		Ecological momentary intervention including self-monitoring	Loaned	Yes	Yes (Messages)	No	Yes (Messages)	2	11	Activity rate 58.0%
SIMPLe (Hidalgo-Mazzei et al. 2016)	Original	Self-monitoring	Personal	Yes	Yes (Messages)	No	Yes (graphical feedback and messages)	1	5	Activity rate: 85.5%
Hidalgo-Mazzei et al. 2018)	Upgraded	Self-Monitoring	Personal	Yes	Yes (Messages)	No	Yes (graphical feedback and messages) °	1	5	Median of use: 60 days. (IQR = 7)
No Name (Wenze et al. 2014)		Self-monitoring	Loaned (PDA)	Yes	Yes (Messages)	No	Yes (messages)	2	16	Activity rate: 91.6%
SIMBA (Beiwinkel et al. 2016)		Self-monitoring	Loaned	Yes	No	Yes (Physical activity and social communication)	No	1	2	55.7%^c^

*IQR* Inter quartile range, *PDA* Personal digital assistant

^a^Only one session a day taken in account for adherence

^b^Undefined adherence characteristics

^c^Calculated relative to active patients only addition of a weekly complementary session

### Adherence characteristics

#### Activity rate

Seven studies calculated an activity rate, ranging from 58.0 to 91.6% (Depp et al. 2015; Wenze et al. 2014, 2016; Faurholt-Jepsen et al. 2020; Hidalgo-Mazzei et al. 2016; Tsanas et al. 2016; Schwartz et al. 2016). Among these seven studies, 6 calculated an activity rate relative to all patients and 1 relative to active patients only (Tsanas et al. 2016).

Schwartz et al. specifically compared the activity rates of the studied app in BD and healthy control groups and did not find a significant difference (79% in the BD group and 71% in the healthy control group, p = 0.22) (Schwartz et al. 2016).

Three studies researched factors influencing the activity rate (Depp et al. 2015; Wenze et al. 2014; Hidalgo-Mazzei et al. 2016). Two studies evaluated the effect of age on the activity rate and failed to find any correlation (Depp et al. 2015; Hidalgo-Mazzei et al. 2016). The third found a trend for a positive association between depressive symptoms and the activity rate (*r *(Ben-Zeev et al. 2013)  = 0.5, p = 0.06) (Wenze et al. 2014).

#### Completion rate

Only one study focused on the number of study days with a fully completed session and found a completion rate of 81.8% (Til et al. 2020). Its primary objective was to understand how to best engage BD patients in the self-monitoring of their symptoms. The authors compared the monitoring of BD patients with active (self-monitoring) and passive (using an activity tracker) data. As the app offered twice daily sessions, the completion rate was defined as logging at least six of 12 symptoms so the data can be compared to apps offering only one daily session. For the activity tracker, the completion rate was measured as the percent of study days with at least 12 h of activity tracking. There was no statistical difference (p = 0.75) in the completion rate between the two groups (active or passing monitoring). Furthermore, to assess the impact of face-to-face reviewing of monitoring, about 50% of participants in both groups were randomly assigned to review their recorded symptoms weekly with an interviewer. Statistical analysis revealed that reviewing recorded symptoms did not significantly improve completion rates (p > 0.80).

#### Median of use and retention rate

Hidalgo-Mazzei et al. studied the median of use and retention rate of the SIMPLe app in two studies, the first evaluating the original version (Hidalgo-Mazzei et al. 2016) and the second an upgraded version (Hidalgo-Mazzei et al. 2018).

With the original app, the mean duration of use was 77 days (SD: 26.2). The number of active patients decreased progressively from 46 (94%) after a month to 40 (82%) after 2 months and to 36 after 3 months (end of the study), giving a 74% retention rate. Regarding predictive factors, a high total Functional Assessment Short Test score and more years of smartphone usage were found to be variables weakly related to the retention rate (p = 0.02 and p = 0.04, respectively).

For the upgraded version, the median of use was 2 months (IQR: 7) and the retention rate was 33.8% at 6 months (end of the study). The mean numbers of users dropping app use each month was 23.3, with almost one-third (n = 70, 34.8%) dropping out during the first month. After 6 months, more than 30% of users regularly interacted with the SIMPLe app. Furthermore, increased age was identified as a factor significantly increasing the odds of retention (OR = 1.0, p < 0.001, CI 95% = 0.0092–0.033) (Hidalgo-Mazzei et al. 2018).

Median of use reported for the MONARCA app was 310 days (IQR: 189-437) in a study that investigated differences between BD type 1 and BD type two patients (Faurholt-Jepsen et al. 2015). With an upgraded version, Stanislaus et al. 2020 reported a median of use of 107 days (IQR: 49-206) for BD patients and 84 days (IQR: 42-121) for healthy controls. In both groups, 80% of participants stayed active after a month (Stanislaus et al. 2020).

#### Undefined adherence characteristics

Three studies did not provide any definition for the adherence characteristics reported (Faurholt-Jepsen et al. 2014, 2015; Beiwinkel et al. 2016). In a pilot study using the SIMBA app, Beiwinkel et al. reported a “compliance rate” of 55.7% (Beiwinkel et al. 2016).

Studying the MONARCA apps, Faurholt-Jepsen et al. reported an “adherence rate” of 88% in a pilot study (Faurholt-Jepsen et al. 2014) and a “mean adherence among time” of 93% in an observational study (Faurholt-Jepsen et al. 2015).

## Discussion

Despite a high number of studies selected at the full article level (118), only 13 possessed the requested inclusion criteria. In particular, 39 eligible studies did not provide any information on adherence characteristics to the app. Among the included studies, study length, sample size and definition of measure of adherence were heterogeneous. Seven studies calculated an activity rate, one study calculated a completion rate, three studies provided a median of use or a retention rate and three studies provided undefined adherence characteristics. Activity rates were disparate, ranging from 58 to 91.6%; these were quite high, with five out of seven being over 70%.

Interestingly, the study that paid patients according to the time spent in the study had one of the lowest activity rates (65%) and the two studies paying patients for each completed session displayed the lowest and the highest activity rates of this review (58% and 91.6%, respectively). These results were in accordance with a meta-analysis finding no difference between paid vs. unpaid patients in their adherence to apps (Torous et al. 2020).

Our main findings should be compared to medication adherence in BD which is a pervasive issue in this chronic disease. The study of medication adherence in BD involves the same difficulties that we encountered in this review, especially heterogeneity in adherence descriptions and measures (Tueller et al. 2016; Greene et al. 2018; Averous et al. 2018). As described in several reviews, mean medication adherence in BD ranges from 50 to 80% with good adherence being an exception (Levin et al. 2016; Chakrabarti 2016). Regarding prevalence, 12-month treatment rates are estimated to be between 45 and 51% (Greene et al. 2018). Many predictive factors for poor medication adherence have been reported, the most common being health beliefs and attitudes to medications (Greene et al. 2018; Averous et al. 2018). Additionally, patients’ attitudes are influenced by several elements such as their family members’ attitude to medication, their knowledge of the illness and their relationship with their clinician (Chakrabarti 2016). This knowledge about medication adherence should be used to guide adherence research in the field of mobile health, both in terms of its predictive factors and ways to improve it.

If the adherence characteristics to the apps found in our review are quite similar to the rates of adherence to treatments found in BD patients, we have to keep in mind that our results are surprisingly high knowing the difficulties encountered by apps to stay attractive over time (Fleming et al. 2018; Baumel et al. 2019). Several aspects could be considered to explain these results.

First, we must recall that a research protocol does not wholly reflect the “real world” conditions; this is particularly true when it comes to mental health and non-drug therapeutic tools. The frame provided by a study, bringing high structuring of participants, is a known adherence-promoting element (Baumel et al. 2019; Ebert and Baumeister 2017). This is confirmed by the studies focusing on the “real world” use of apps that highlighted poor adherence (Bauer et al. 2020; Fleming et al. 2018; Baumel et al. 2019). The included studies in this review are no exception to this bias and it is very likely that the described apps would be less used in real world conditions, without the clinical frame offered by the study protocol. Interestingly, the real-world implementation observational study of the SIMPLe app did not offer clinical interviews to participants but carried out assessments through email only (Hidalgo-Mazzei et al. 2018). This method of evaluation could reduce the bias created by clinical frames in the measure of adherence in studies. However, the lack of face-to-face contact with the patient in fully online study seems to impact negatively the adherence with the app (retention rate of 30% at the end of the study).

Secondly, some of the reviewed studies presented adherence characteristics relative to active patients only. This method of evaluation excludes the notion of retention and so does not allow a reliable assessment of adherence and provides higher rates than if calculated relative to all participants. It provides information about global activity and could help to determine the most suitable rhythm of evaluation for the app (once or twice a day, once a week), but fails to consider participants that discontinued their use of the app in question. Thus, a 93% activity rate calculated on active patients can only hide a 70% drop-out rate.

Another reason for the high adherence characteristics found in our review could be the age of participants. The mean age among studies was 39.9 years (IQR: 35.6-44.3). This mean age and limited dispersion exclude the elderly and could hide the famous “generation gap”. Older adults are commonly expected to have difficulties acclimating to new technologies such as smartphone apps (Grossman et al. 2020). This theory tends to be refuted (Bennett et al. 2008) and progressively gives way to the conception of older adults as a heterogeneous population regarding their abilities to use new technologies (Jayasinghe et al. 2019; Mitzner et al. 2010). A review of mobile technology among older adults even suggested that, far from being refractory to mobile intervention, they generally show a desire to engage with these technologies to monitor and improve health conditions (Kuerbis et al. 2017). However, there are still factors that inhibit their use of mobile technologies such as functional capacities, cognitive changes, deterioration of fine motor coordination, visual impairment and previous experience with or exposure to smartphones (Mitzner et al. 2010). In our review, no study found any negative correlation between an older age and adherence; one even identified a positive correlation between increased age and retention (Hidalgo-Mazzei et al. 2016). Further studies should therefore specifically focus on the implication of age in adherence to determine whether there is a generation gap regarding the use of mobile technology in mental health care.

Finally, adherence characteristics can be inflated because of publication bias. In a meta-analysis focusing on clinical trials of smartphone apps for depressive symptoms, Torous et al. reported a drop-out rate of 26.2% (Torous et al. 2020). When they adjusted this estimation to publication bias, it rose to 47.8%. Finding unimportant or negative results is known to be one of the main reasons for non-publication of completed studies (Song 2013). Considering the development of an app, low adherence characteristics could be read as a negative outcome and so lead to the non-publication of results. This element should therefore be taken into account for the interpretation of published adherence characteristics.

### Future research

The word “adherence” is a generic term covering many different notions. In articles, it is interchangeably used with the terms “engagement” or “uptake”. The most relevant information highlighted by this review was the high number of studies that did not provide any information on adherence characteristics and study heterogeneity regarding method and provided data. This lack of a standardised measure of adherence to smartphone apps has been mentioned in several systematic reviews or meta-analyses focusing on smartphone app interventions for severe mental illness and depression (Kerst et al. 2019; Rathbone and Prescott 2017; Berry et al. 2016). As mentioned in the introduction, adherence is a key factor in the development of apps for BD. Its assessment with standardised methods could allow a meta-analysis to be carried out and statistical determinations of its predictive factors to be made. For further studies involving the use of an app, it could be of interest to systematically assess and report the following items, regardless of the primary study outcomes: activity rate, completion rate, median of use and retention rate. A focus on which features of the apps are more likely used (self-assessment, psychoeducation) could also be of interest. In addition, the collection of patients’ feedback regarding the studied app, using Likert scales or a qualitative design, could be potential ways to upgrade apps following a user-centred approach. Beyond these measures, apps adherence should be the object of a broad expert systematic consensus in the scientific community to define all its aspects. In particular, threshold determining a good or acceptable adherence should be defined and its parameters should be standardised. Such a consensus is essential to compare studies with each other and to allow improvement in apps adherence. Furthermore, the access to app usage data is technically simple and should allow these standards to be easily applied to all studies.

Finally, the development of guided apps (i.e., apps used under the supervision of a professional) over self-help apps (i.e., apps freely downloaded and used by patients) should be preferred (Baumel et al. 2019; Ebert and Baumeister 2017). Indeed, several studies focusing on self-help apps found patient reluctance toward these tools (Kerst et al. 2019; Fuller-Tyszkiewicz et al. 2018; Stiles-Shields et al. 2017) and a lower adherence compared to guided one (Torous et al. 2020; Eysenbach 2005; Titzler et al. 2018; Cuijpers et al. 2017; Linardon et al. 2019). Furthermore, the better adherence to apps in research protocols compare to real world highlights the essential role of face-to-face meetings in mental healthcare (Baumel et al. 2019; Ebert and Baumeister 2017). If they are able to facilitate access to psychiatric care to the largest number of individuals (BinDhim et al. 2015; Ramos et al. 2019; Cheng et al. 2016), self-help apps should be restricted to screening tools, and their main purpose should be to guide patients toward medical care. Therapeutic apps should be fully integrated in therapy and be closely monitored by therapists. In addition to increasing adherence, their integration could reinforce the patient-therapist link and offer to the therapist better knowledge of their patient’s clinical state, particularly during the in-between visits period.

## Limitations

The heterogeneity of apps, study designs, clinical approaches and participant populations analysed limited the generalisation of our results. The lack of standardised reports of adherence forced us to compare similar but not identical data. No study reported on how many features of the apps were used. Furthermore, five studies included were rated with poor quality because of small sample size. They were either pilot study or feasibility studies. Finally, this review included only apps that have been studied in a research protocol. To enlarge the scope of this work, the assessment of adherence to apps available on app stores would be of great interest to future work.

## Conclusion

In total, maintaining adherence over time is a key factor to increase apps implementation in daily care and to improve quality of care. This review highlights the overwhelming lack of documentation of adherence to apps in the actual literature, as well as the great disparity of its definition and measures. These results encourage the establishing of a systematic standard evaluation of adherence in study protocols involving smartphone app interventions.

## Supplementary Information

**Additional file1: Table S1.** Quality assessment of RCT using the national institutes of health (NIH) quality assessment tool for controlled intervention studies

**Additional file2: Table S2.** Quality assessment of longitudinal studies using the national institutes of health (NIH) quality assessment tool for observational cohort and cross-sectional studies

**Additional file 3: Table S3.** Quality assessment of studies with no control group using the national institutes of health (NIH) quality assessment tool for before-after (pre-post) studies with no control group

## Data Availability

The datasets used and/or analysed during the current study are available from the corresponding author on reasonable request.

## Abbreviations

BD: Bipolar disorder
CARS: Clinician-administered rating scale for mania
IQR: Inter Quartile range
PDA: Personal digital assistant
QIDS: Quick inventory of depressive symptoms
SD: Standard deviation
