# Supplementary material for: Patients’ adherence to smartphone apps in the management of bipolar disorder: a systematic review
Source: Int J Bipolar Disord. 2021 Jun 3;9:19. doi: 10.1186/s40345-021-00224-6 (PMC8175501; doi:10.1186/s40345-021-00224-6)
Supplement: Supplementary file 1 — Additional file1: Table S1. Quality assessment of RCT using the national institutes of health (NIH) quality assessment tool for controlled intervention studies [file 40345_2021_224_MOESM1_ESM.pdf]

Additional File 1 : Quality Assessment of RCT using the National Institutes of Health (NIH) Quality Assessment Tool for Controlled Intervention Studies

Major Components	Studies		
	Van Til et al.; 2020, (30)	Faurholt-Jepsen et al.; 2020, (27)	Depp et al.; 2015; (7)
1. Was the study described as randomized, a randomized trial, a randomized clinical trial, or an RCT?	Yes	Yes	Yes
2. Was the method of randomization adequate (i.e., use of randomly generated assignment)?	Yes	Yes	Yes
3. Was the treatment allocation concealed (so that assignments could not be predicted)?	NR	Yes	Yes
4. Were study participants and providers blinded to treatment group assignment?	No	No	No
5.Were the people assessing the outcomes blinded to the participants' group assignments?	No	Yes	Yes
6. Were the group similar at baseline on important characteristics that could affect outcomes (e.g., demographics, risk factors, co-morbid conditions)?	Yes	Yes	Yes
7. Was the overall drop-out rate from the study at endpoint 20% or lower of the number allocated to treatment?	Yes	Yes	Yes
8. Was the differential drop-out rate (between treatment groups) at endpoint 15 percentage points or lower?	Yes	Yes	Yes
9. Was there high adherence to the intervention protocols for each treatment group?	Yes	No	No
10. Were other interventions avoided or similar in the groups (e.g., similar background treatments)?	Yes	Yes	Yes
11. Were outcomes assessed using valid and reliable measures, implemented consistently across all study participants?	Yes	Yes	Yes
12. Did the authors report that the sample size was sufficiently large to be able to detect a difference in the main outcome between groups with at least 80% power?	No	Yes	No
13. Were outcomes reported or subgroups analyzed prespecified (i.e., identified before analyses were conducted)?	Yes	Yes	Yes
14. Were all randomized participants analyzed in the group to which they were originally assigned, i.e., did they use an intention-to-treat analysis?	NR	Yes	No
Quality Rating	Fair	Good	Good
Additional Comments (If Poor, please state why):			

RCT: Randomized Controlled Trial; NR: Not Reported; NA : Not Applicable
