# Supplementary material for: Patients’ adherence to smartphone apps in the management of bipolar disorder: a systematic review
Source: Int J Bipolar Disord. 2021 Jun 3;9:19. doi: 10.1186/s40345-021-00224-6 (PMC8175501; doi:10.1186/s40345-021-00224-6)
Supplement: Supplementary file 2 — Additional file2: Table S2. Quality assessment of longitudinal studies using the national institutes of health (NIH) quality assessment tool for observational cohort and cross-sectional studies [file 40345_2021_224_MOESM2_ESM.pdf]

Additional File 2: Quality Assessment of longitudinal studies using the National Institutes of Health (NIH) Quality Assessment Tool for Observational Cohort and Cross-Sectional Studies.

**Major Components**	**Studies**		
	Stanislaus et al.; 2020,	Faurholt-Jepsen et al.; 2015;	Tsanas et al.; 2016, (34)
1. Was the research question or objective in this paper clearly stated?	Yes	Yes	Yes
2. Was the study population clearly specified and defined?	Yes	Yes	No
3. Was the participation rate of eligible persons at least 50%?	Yes	Yes	CD
4. Were all the subjects selected or recruited from the same or similar populations (including the same time period)? Were inclusion and exclusion criteria for being in the study prespecified and applied uniformly to all participants?	Yes	Yes	Yes
5. Was a sample size justification, power description, or variance and effect estimates provided?	No	Yes	No
6. For the analyses in this paper, were the exposure(s) of interest measured prior to the outcome(s) being measured?	Yes	Yes	Yes
7. Was the timeframe sufficient so that one could reasonably expect to see an association between exposure and outcome if it existed?	Yes	Yes	Yes
8. For exposures that can vary in amount or level, did the study examine different levels of the exposure as related to the outcome (e.g., categories of exposure, or exposure measured as continuous variable)?	Yes	NA	NA
9. Were the exposure measures (independent variables) clearly defined, valid, reliable, and implemented consistently across all study participants?	Yes	Yes	Yes
10. Was the exposure(s) assessed more than once over time?	NA	NA	NA
11. Were the outcome measures (dependent variables) clearly defined, valid, reliable, and implemented consistently across all study participants?	No	Yes	Yes
12. Were the outcome assessors blinded to the exposure status of participants?	No	No	No
13. Was loss to follow-up after baseline 20% or less?	NA	NA	NA
14. Were key potential confounding variables measured and adjusted statistically for their impact on the relationship between exposure(s) and outcome(s)?	Yes	Yes	Yes
Quality Rating	Fair	Fair	Fair
Additional Comments (If Poor, please state why):			

CD: Cannot determined; NA: Not applicable
