# Supplementary material for: Patients’ adherence to smartphone apps in the management of bipolar disorder: a systematic review
Source: Int J Bipolar Disord. 2021 Jun 3;9:19. doi: 10.1186/s40345-021-00224-6 (PMC8175501; doi:10.1186/s40345-021-00224-6)
Supplement: Supplementary file 3 — Additional file 3: Table S3. Quality assessment of studies with no control group using the national institutes of health (NIH) quality assessment tool for before-after (pre-post) studies with no control group [file 40345_2021_224_MOESM3_ESM.pdf]

Additional File 3: Quality Assessment of studies with no control group using the National Institutes of Health (NIH) Quality Assessment Tool for Before-After (Pre-Post) Studies with no Control Group.

**Major Components**	**Studies**						
	Hidalgo-Mazzei et al.; 2018, (32)	Wenze et al.; 2016; (29)	Hidalgo-Mazzei et al.; 2016;(31)	Wenze et al. 2014, (8)	Beiwinkel et al; 2016;(33)	Schwartz et al., 2016, (36)	Faurholt -Jepsen et al.; 2014, (28)
1. Was the study question or objective clearly stated?	Yes	Yes	Yes	Yes	Yes	Yes	Yes
2. Were eligibility/selection criteria for the study population prespecified and clearly described?	Yes	No	Yes	No	Yes	No	Yes
3. Were the participants in the study representative of those who would be eligible for the test/service/intervention in the general or clinical population of interest?	Yes	Yes	Yes	Yes	Yes	Yes	Yes
4. Were all eligible participants that met the prespecified entry criteria enrolled?	NA	No	Yes	No	No	No	No
5.Was the sample size sufficiently large to provide confidence in the findings?	No	No	No	No	No	No	No
6. Was the test/service/intervention clearly described and delivered consistently across the study population?	Yes	Yes	Yes	Yes	Yes	Yes	Yes
7. Were the outcome measures prespecified, clearly defined, valid, reliable, and assessed consistently across all study participants?	Yes	Yes	Yes	Yes	Yes	Yes	Yes
8. Were the people assessing the outcomes blinded to the participants' exposures/interventions?	No	No	No	No	No	No	No
9. Was the loss to follow-up after baseline 20% or less? Where those lost to follow-up accounted for in the analysis?	No	No	No	Yes	Yes	Yes	Yes
10. Did the statistical methods examine changes in outcome measures from before to after the intervention? Were statistical tests done that provided p values for the pre-to-post changes?	Yes	Yes	Yes	Yes	Yes	Yes	Yes
11. Were outcome measures of interest taken multiple times before the intervention and multiple times after the intervention (i.e., did they use an interrupted time-series design)?	No	No	No	No	No	NA	No
12. If the intervention was conducted at a group level (e.g., a whole hospital, a community, etc.) did the statistical analysis take into account the use of individuals-level data to determine effects at the group level??	NA	NA	NA	NA	NA	NA	NA
Quality Rating	Fair	Poor	Fair	Poor	Poor	Poor	Poor
Additional Comments (If Poor, please state why):		Small sample		Small sample	Small sample	Small Sample	Small sample

NA: Not Applicable
